# CRB3 and the FERM protein EPB41L4B regulate proliferation of mammary epithelial cells through the release of amphiregulin

**DOI:** 10.1371/journal.pone.0207470

**Published:** 2018-11-15

**Authors:** Stephanie J. Walker, Laura M. Selfors, Ben L. Margolis, Joan S. Brugge

**Affiliations:** 1 Department of Cell Biology, Harvard Medical School, Boston, Massachusetts, United States of America; 2 Department of Internal Medicine, University of Michigan, Ann Arbor, Michigan, United States of America; Hillsdale College, UNITED STATES

## Abstract

Numerous observations have suggested a connection between the maintenance of cell polarity and control of cell proliferation; however, the mechanisms underlying these connections remain poorly understood. Here we found that ectopic expression of CRB3, which was previously shown to restore tight junctions and membrane polarity in MCF-10A cells, induced a hyperproliferative phenotype, with significantly enlarged acini in basement membrane culture, similar to structures induced by expression of proliferative oncogenes such as cyclinD1. We found that CRB3-induced proliferation is epidermal growth factor (EGF)-independent and occurs through a mechanism that involves secretion of the EGF-family ligand, amphiregulin (AREG). The increase in AREG secretion is associated with an increase in the number and size of both early and late endosomes. Both the proliferative and endocytic phenotypes associated with CRB3 expression require the FERM-binding domain (FBD) but not the PDZ-binding domain of CRB3, arguing that this proliferative phenotype is independent of the PDZ-dependent polarity signaling by CRB3. We identified the FBD-containing protein, EPB41L4B, as an essential mediator of CRB3-driven proliferation and observed that the CRB3-dependent changes in endocytic trafficking were also dependent on EPB41L4B. Taken together, these data reveal a previously uncharacterized role for CRB3 in regulating proliferation in mammalian cells that is associated with changes in the endocytic trafficking machinery.

## Introduction

Glandular epithelial cells, such as those in the mammary gland, are organized into secretory structures with an epithelial monolayer that surrounds a hollow lumen and distinct morphological features, such as specialized cell–cell contacts and a polarized distribution of organelles and membrane proteins. A common feature of cancers of epithelial origin is the disruption of normal cell polarity and a loss of the higher order organization of the epithelial tissue. Alteration of proteins involved in establishment or maintenance of epithelial polarity is often observed in carcinomas. In addition to their known functions in the establishment and maintenance of cell polarity, many of these proteins regulate other processes relevant to tumorigenesis, such as proliferation and apoptosis [1].

Our laboratory has used the MCF-10A mammary epithelial cell line, which is competent to form mammary acini in three-dimensional (3D) culture, as a model system to study the normal morphogenesis of glandular epithelial tissues and how disruption of this morphogenetic program contributes to tumorigenesis. In normal acinar development, a single epithelial cell proliferates to form a disorganized cluster of cells. Subsequently, the outer cells in contact with the extracellular matrix establish an axis of polarity and undergo proliferative suppression, while the inner cells are deprived of matrix attachment and undergo apoptosis forming the hollow lumen. The interplay between cell proliferation and epithelial architecture in this system is complex. Proliferative oncogenes, such as cyclinD1, induce hyperproliferation of the outer cells leading to enlarged structures [2]. In contrast, expression of the ErbB2 oncogene not only leads to increased proliferation but also causes a disruption in the normal architecture, producing multi-acinar structures [3]. How the architecture of the epithelial tissue is coordinated with proliferation and how these processes impact each other during tumorigenesis is not well understood. Whether disruption of epithelial architecture plays a causative role in cancer progression or if this loss of organization is simply a secondary phenotype caused by the transformed state has emerged as an important topic of current research in the field.

In 3D culture, the MCF-10A mammary epithelial acini develop an axis of polarity in which the basement membrane is deposited and organized at the basal surface of the outer acinar cells and the Golgi orient to the apical side; however, the outer cells do not develop a discrete apical surface or tight junctions. We initially became interested in understanding the role of CRB3 as a regulator of polarity in mammary epithelial cells, based on reports that CRB3 expression stabilized tight junction formation in MCF-10A mammary cells [4]. The three mammalian Crumbs proteins, CRB1-3, have known functions in apical membrane formation and polarity establishment, with CRB3 specifically playing this role in epithelial cells by virtue of its specific expression in this cell lineage. The CRB3 intracellular sequence contains two motifs that are conserved within *Drosophila* Crumbs and are identified in all mammalian CRB isoforms: a juxtamembrane FERM (band 4.1/ezrin/radixin/moesin) binding domain (FBD) and a carboxy-terminal PDZ (PSD-95/Dlg/ZO1) binding domain with the sequence ERLI [4]. The PDZ domain has a well-established role in the CRB-dependent regulation of cell polarity through a mechanism that involves direct interactions with the Pals1/PATJand PAR/aPKC complexes [5]. Recent studies have suggested a role for CRB3 in regulating cancer stem cell properties of mammary epithelial cells via Pals1/PATJ [6, 7]. The function of the FBD is not as well defined but is thought to link the apical CRB complex to the actin cytoskeleton [5].

In our effort to understand the interplay of cell polarity and proliferation of mammary epithelial cells, we made the unexpected observation that expression of the polarity protein CRB3 confers a proliferative phenotype in mammary epithelial cells. In the work presented here, we characterized the mechanisms underlying this CRB3-dependent phenotype. Ectopic expression of CRB3 in MCF-10A cells induced a hyperproliferative phenotype in acini and supported epidermal growth factor (EGF)-independent proliferation in both monolayer and basement membrane cultures, through a mechanism that involves secretion of the EGF-family ligand, amphiregulin (AREG). The increase in AREG secretion was associated with an increase in the number and size of both early and late endosomes. Both the proliferative and endocytic phenotypes associated with CRB3 expression required the FERM-binding domain (FBD) but not the PDZ-binding domain of CRB3, arguing that this proliferative phenotype is independent of the PDZ-dependent polarity signaling by CRB3. Expression profiling and a directed siRNA screen identified the FBD-containing protein, EPB41L4B, as an essential mediator of CRB3-driven proliferation and CRB3-dependent changes in endocytic trafficking. Bioinformatics analyses in tumor cell lines and patient samples confirm a positive correlation in CRB3 and EPB41L4B expression. Taken together, these data reveal a previously uncharacterized role for CRB3 in regulating proliferation in mammalian cells that is associated with changes in the endocytic trafficking machinery.

## Materials and methods

### Antibodies, plasmids, and reagents

Antibodies used for immunofluorescence of acinar cultures have been described previously [8]. The following antibodies were used for immunoblotting: phospho-EGFR (Y845) (Cell Signaling Technology, 2231), EGFR (Cell Signaling Technology, 2232), phospho-ERK1/2 (Invitrogen, 44-680G), GAPDH (Ambion, AM4300), γ-tubulin (Abcam, ab6046), HA-tag, clone 6E2 (Cell Signaling Technology, 2367), and c-myc, clone 9E10 (Santa Cruz, sc-40). The following antibodies were used for immunofluorescence: myc, clone 4A6 (Millipore, 05–724), amphiregulin, clone 31221 (R&D Systems, MAB262), FITC-CD63 (Pierce, MA1-19602), FITC-EEA1 (BD Biosciences, 612006), and FITC-LAMP-2, CD107b (ThermoFisher Scientific, RMCD107B01). All EGFR ligand-neutralizing antibodies were purchased from R&D Systems. The monoclonal antibody C225 was provided by H. Steve Wiley (Pacific Northwest National Laboratory). A rabbit polyclonal antibody to the carboxy-terminus of the human CRB3 sequence (VGARVPPTPNLKLPPEERLI) was synthesized (QCB, Hopkinton, MA) based on a previously published antibody sequence [9]. The pNG retroviral vectors encoding human amino-terminal myc-CRB3 and CRB3 mutants [4] were obtained from Ben Margolis (University of Michigan Medical School). The tagged CRB3 constructs were digested with Not1-XhoI and subcloned into the pLNCX2 retroviral vector (Clontech) with compatible sites (Not1-SalI). Murine EPB41L4B with an amino-terminal hemagluttinin (HA) epitope was synthesized by GenScript and cloned into pUC57 with BamHI and HindIII flanking sites. An additional HA tag was inserted at an internal XmaI site using a linker oligo encoding the HA tag with XmaI sites on both ends. The doubly HA-tagged EPB41L4B was subcloned into pLPCX (Clontech) using the BglII-HindIII site on the destination vector. All pooled and individual siRNA oligonucleotides were purchased from Dharmacon, and all other oligonucleotides were purchased from IDT.

### Cell culture, virus production, and stable cell lines

MCF-10A cells were cultured as described [8]. VSV-G pseudotyped retroviruses were generated, and MCF-10A cells were infected and selected as described [8] using a VSV-GPG producer line [10]. VSV-G pseudotyped lentiviruses were produced by co-transfection of pLKO hairpin vectors with accessory plasmids in 293T cells. Hairpin-encoding lentiviruses were used to infect MCF-10A cells or superinfect CRB3-expressing cells, and stable cell lines were obtained by selection with puromycin (2 μg/ml) or dual selection with puromycin (2 μg/ml) and G418 (300 μg/ml), respectively.

### 3D morphogenesis assays

Cells were cultured in reconstituted growth factor reduced basement membrane (Matrigel; BD Biosciences, San Jose, CA) and processed for immunofluorescence and confocal microscopy as described [8]. Acinar diameters were measured using Image J (National Institutes for Health). For cell cycle analysis of 3D cultures, cells were harvested from Matrigel cultures by trypsinization for 30 minutes at 37°C, fixed in 75% ethanol, treated with RNase A (0.25 mg/ml), stained with propidium iodide (10 μg/ml), and analyzed on a FACSCalibur flow cytometer (BD Biosciences) for DNA content. Data were analyzed using CellQuest (BD Biosciences).

### EGF-independent proliferation assays

For EGF-independent proliferation in 3D, cells were plated and cultured in the absence of exogenous EGF. For EGF-independent proliferation in monolayer, MCF-10A cells were seeded at 5 x 10^4^ cells in assay medium in the absence of exogenous EGF and counted on the days indicated [8]. For experiments with ligand neutralizing antibodies, the antibodies were added at the time of EGF withdrawal at the following concentrations: HB-EGF, 5 μg/ml; AREG, 1 μg/ml; TGFα 0.5 μg/ml; IgG 1 μg/ml; and mAb C225 10 μg/ml. Conditioned medium was collected from cells after 48 hours in assay medium without EGF and filtered through a 0.2 μm filter prior to addition to wild-type MCF-10A cells. For all cell number quantification experiments, cells were harvested by trypsinization and counted on a hemocytometer.

### AREG ELISA

Media was collected from cells after 24 hours in EGF-free assay medium or at the times indicated and quantified using a human AREG ELISA according to the manufacturer’s protocol (R&D Systems).

### Gene expression analysis by microarray and real-time quantitative RT-PCR (RT-qPCR)

For microarray analysis, total RNA was isolated in triplicate from proliferating monolayers (70% confluent) of vector control and CRB3-expressing MCF-10A cells using TRIzol (Invitrogen) and the RNeasy MinElute Cleanup Kit (Qiagen). RNA samples were then processed by the Biopolymers Facility at Harvard Medical Facility for hybridization to Human Genome U133 Plus 2.0 gene chips (Affymetrix, Santa Clara, CA). The experiment was carried out in triplicate. Background correction and normalization were performed using the Robust Multichip Average method implemented in the rma function of R/BioConductor. The differential expression was assessed using the empirical Bayes method implemented in the limma package. The criteria for selecting differentially expressed genes was a false discovery rate (FDR)-corrected p-value of less than 0.05 and a fold change greater than 1.5. The data has been deposited in the GEO database (GSE76610). Microarray enrichment analysis of differentially expressed genes in CRB3-expresssing MCF-10A cells. Bars represent enrichment scores, defined as -log(pValue), of the top pathways identified by GeneGO enrichment analysis (MetaCore, GenGO; Thomson Reuters). The dashed line designates the threshold for statistical significance (p = 0.05). For quantitative RT-PCR, RNA isolated using the TRIzol protocol was subjected to first-strand cDNA synthesis and RT-qPCR as described previously [11]. Samples were normalized to the geometric mean of three housekeeping genes (GAPDH, HINT1, PRDX6) as described [11, 12]. All samples were isolated and measured as biological triplicates. The following primer sequences were used for RT-qPCR:

AREG fwd: 5´-TACTCGGCTCAGGCCATTAT-3´

rev: 5´-AGCCAGGTATTTGTGGTTCG-3´

EPB41L4B fwd: 5´-GCGAAGTGGCTGGAAATGTATG-3´

rev: 5´-GAAGAATGCGTGGTGCTCAAC-3´

GAPDH fwd:5´-AACGTGTCAGTGGTGGACCT-3´

rev: 5´-TCGCTGTTGAAGTCAGAGGA-3´

HINT1 fwd: 5´-GCCTTGCTTTCCATGACATT-3´

rev; 5´-CCTTATTCAGGCCCAGATCA-3´

PRDX6 fwd, 5´-CGTGTGGTGTTTGTTTTTGG-3´

rev, 5´-CTTCTTCAGGGATGGTTGGA-3´

### RNA interference

For siRNA transfections, 25–50 nmol of siRNA were transfected using Oligofectamine according to the manufacturer’s protocol (Invitrogen). Knockdown efficiency was analyzed at 48–72 hours post-transfection by immunoblotting or quantitative PCR. Functional assays (AREG ELISA, immunofluorescence) were carried out 72 hours following siRNA transfection, which included 24 hours of EGF starvation. pLKO.1 puro-based lentiviral shRNA vectors were provided by the RNAi Consortium at the Broad Institute (Cambridge, MA). The EPB41L4B shRNA #1 and #2 were TRCN0000062396 and TRCN0000062397, respectively. The siGENOME SMARTpools and corresponding individual siRNA upgrades (Dharmacon) were as follows: AREG (M-017435-00), EPB41L4B (M-013803-01), EPB41L5 (M-010729-01), PTPN21 (M-009379-02), MPP5/PALS1 (M-009388-00), PARD6A (M-013859-01), PARD6B (M-010681-01), and PARD6G (M-014909-01).

### FERM gene annotation and siRNA screen

To identify genes that may play a role in signaling through the CRB3 FERM-binding domain, we used the Biomart system (http://www.biomARt.org/index.html) to search the human genome for transcripts with an annotated FERM domain (IPR000299). This search identified 226 mRNA transcripts that contain a FERM domain. Using the NIH DAVID Database, these transcripts map to 46 unique validated genes that encode proteins containing a conserved FERM domain. We assembled a panel of Dharmacon SMARTpool siRNA reagents targeting 27 of these genes, focusing on targets with established roles in kinase-mediated signaling and cytoskeletal regulation based on the published literature and functional annotation clustering using NIH DAVID. CRB3-epxressing MCF-10A cells were seeded at 3.8 x 10^3^ cells per well in 96 well plates. 24 h after seeding, Dharmacon SMARTpools for the indicated genes (S1 File) were transfected into the cells as described for siRNA transfection. 24 h post-transfection, the media was replaced with regular growth medium. 48 h post-transfection, the cells were starved for EGF for 24 h, at which time the media was collected and quantified for AREG release using the AREG ELISA. Hits from this primary screen were identified as those for which the average fold change in AREG release was >1.15 (log2 scale). These genes were then rescreened using the individual siRNA upgrades for the Dharmacon SMART pools used in the primary screen.

### Oncomine data mining

Normalized mRNA expression values for CRB3, EPB41L4B, and EPB41L5 were obtained from multiple data sets. Pearson product-moment correlation coefficients and associated p-values were calculated in JMP 7.0 software.

### Immunofluorescence and image acquisition

Acinar structures were immunostained as previously described [8]. Confocal images were acquired on a Nikon TE2000 inverted microscope with a Nikon C1si spectral point scanning confocal head using the Nikon EZ-C1 software. 404nm argon and 488nm diode lasers were used for excitation with 450/35 and 535/50 emission filters, respectively, using either a 40X Plan Fluor 0.13 NA (acini) or a 60X Plan Fluor 1.4 NA (puncta) objective. All phase contrast images and live fluorescent images were acquired using either a 4X Plan Fluor 0.13 NA or a 10X Plan Fluor 0.30 NA objective on a Nikon TE300 manual inverted microscope with an MTI RC300 CCD monochrome camera and ImageJ.

For immunofluorescence of monolayer cultures, cells were fixed in 2% formalin/PBS, permeabilized with 0.02% (w/v) saponin, washed with PBS/200mM glycine, blocked in 1% (w/v) FBS/1% (w/v) BSA, and then stained with the indicated primary antibodies followed by secondary antibody and DAPI staining. Primary and secondary antibody incubations were carried out in the presence of block solution plus saponin. Indirect fluorescent images were acquired on a Nikon 80i upright with a Hamamatsu C8484-03 monochrome camera with MetaMorph acquisition software. All images were converted to TIFF format and processed in Adobe Photoshop or Illustrator for presentation.

### Western blot analyses

Cells were scraped in RIPA lysis buffer containing protease and phosphatase inhibitors and lysed for 10 minutes on ice. Lysates were cleared by centrifugation at 12,000xg at 4°C for 15 min. Cleared lysates were quantified for protein concentration using a BCA assay (Pierce) and equal amounts of protein for each sample were resolved by SDS-PAGE on 4–20% gradient Tris-glycine gels. Samples were transferred to PVDF membranes (Immobilon-P from Millipore), probed with primary and secondary antibodies, and developed using ECL detection.

## Results

### Ectopic CRB3 expression leads to hyperproliferation in MCF-10A

**Fig 1:**
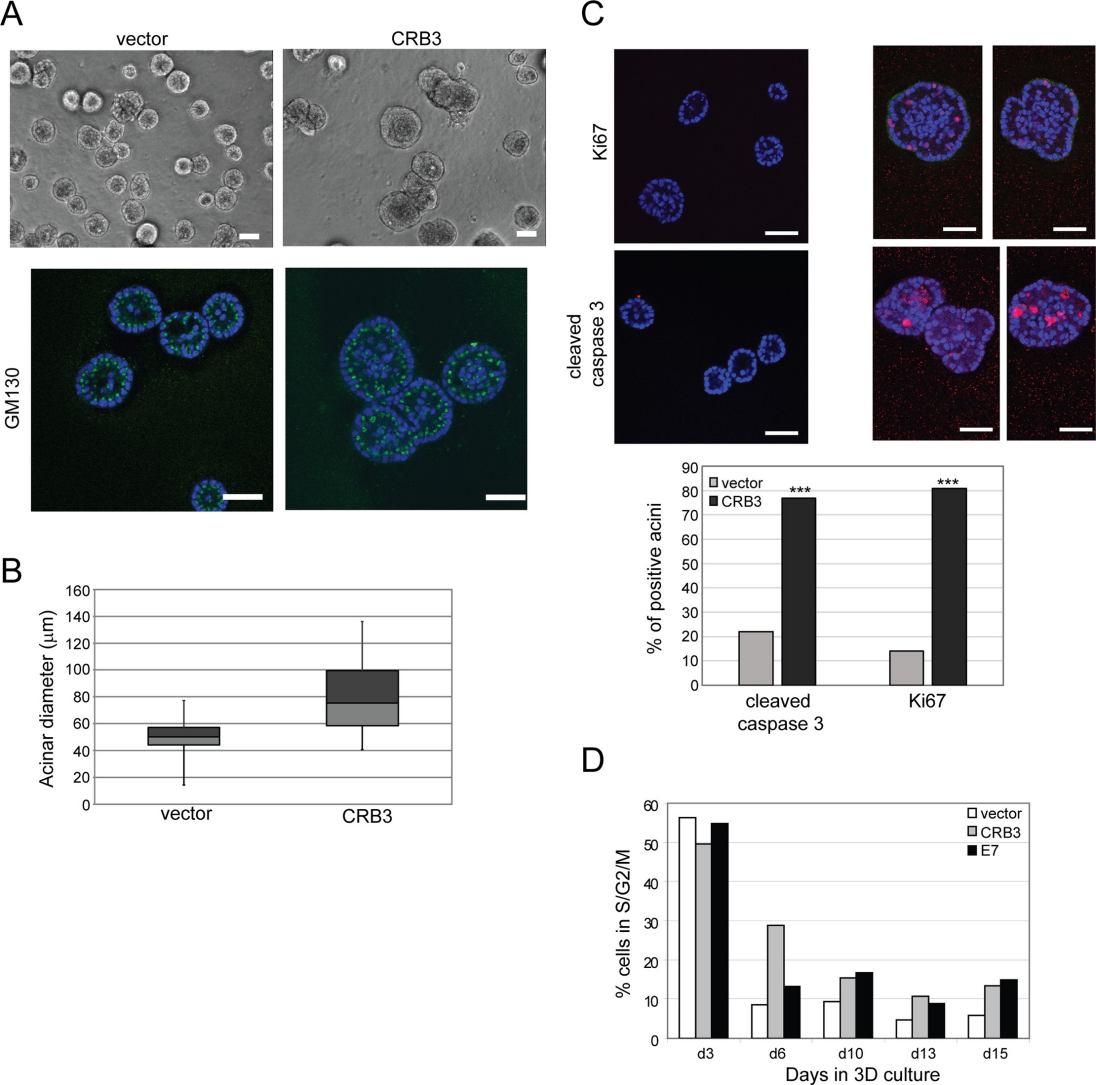
CRB3 expression leads to hyperproliferation and enlarged acini of MCF-10A cells. **(A)** MCF-10A cells expressing CRB3 cells were cultured on Matrigel for 13 days. Representative phase images are shown (top) along with equatorial TOPRO-3-stained (blue) confocal cross sections of structures and immunostained with the Golgi marker, GM130 (green). Representative images of are shown. Scale bars, 50 μm. **(B)** The diameters of acini were measured in Image J using phase images of day 12 cultures, and a boxplot of acinar diameters from seven fields pooled from three independent experiments is shown (top). The light and dark grey boxes represent the lower and upper quartiles, respectively, with the median dividing the two boxes. The whiskers represent the minimum and maximum data points. The average acinar diameters +/- SEM were 49.4 +/- 1.4 μm and 79.4 +/- 2.9 μm for vector and CRB3 acini, respectively (p = 2.2 x 10^−15^). **(C)** MCF-10A cells expressing CRB3 were cultured on Matrigel for 13 days and equatorial TOPRO-3-stained confocal cross sections of structures immunostained with Ki67 (top) or cleaved caspase 3 (middle) are shown. Ki67, cleaved caspase 3 in red; TOPRO-3 (nuclei) in blue. Scale bars, 50 μm. The percent of acini positive for either Ki67 or cleaved caspase 3 were quantified from acini pooled from three independent experiments (bottom). (Ki67: vector, n = 27; CRB3, n = 39; cleaved caspase 3: vector, n = 21, CRB3, n = 27; ***p< 0.001 by Fisher’s exact test). **(D)** Single cell suspensions of the indicated cell types were prepared from cultures on the days indicated, labeled with propidium iodide, and analyzed by flow cytometry to quantify the percentage of cells with DNA content corresponding to the S/G2/M phases of the cell cycle. Flow cytometry data shown is from a representative experiment of two biological replicate experiments.

To investigate the role of polarity proteins in mammary acinar morphogenesis, an amino-terminal myc-tagged variant of the polarity regulator, CRB3, was stably expressed in MCF-10A mammary epithelial cells [4]. Interestingly, CRB3-expressing MCF-10A cells formed acini that were on average 30 μm (approximately 2-fold) larger in diameter than normal acini (Fig 1A and 1B). Importantly, the enlarged CRB3-expressing acini maintained a normal axis of polarity as evidenced by the orientation of the Golgi apparatus toward the apical surface of the acini (Fig 1A), demonstrating that the polarized architecture of MCF-10A acini was not disrupted in the larger structures induced by CRB3 expression.

During the development of normal mammary acini, cells attached to the basement membrane undergo proliferative suppression and cells within the presumptive lumen are cleared to form a hollow sphere. The enlarged CRB3-expressing acini displayed the hallmarks of MCF-10A hyperproliferative structures, a phenotype observed in response to expression of oncogenes that allow escape from proliferative suppression, such as cyclinD1 and human papilloma virus E7 [2]. Specifically, CRB3-expressing acini showed an increase in Ki67 staining, a marker for proliferating cells, on day 13 of acinar culture when normal acini are growth arrested. The higher proliferation observed in the CRB3-expressing acini was balanced by increased apoptosis as marked by cleaved caspase 3 staining in the presumptive lumen (Fig 1C). Furthermore, acini from CRB3-expressing cells maintain a higher percentage of cells in the S/G2/M stages of the cell cycle beyond the time when normal acini have undergone proliferative suppression, a phenotype similar to acini expressing the HPV viral oncoprotein E7 [2] (Fig 1D). Taken together, these results support the unexpected finding that ectopic expression of the polarity protein CRB3 allows MCF-10A to escape proliferative suppression during morphogenesis in 3D culture but does not suppress luminal apoptosis, leading to the generation of enlarged, hollow, hyperproliferative structures.

### CRB3 expression leads to epidermal growth factor-independent proliferation through secretion of the EGF receptor ligand, amphiregulin

**Fig 2:**
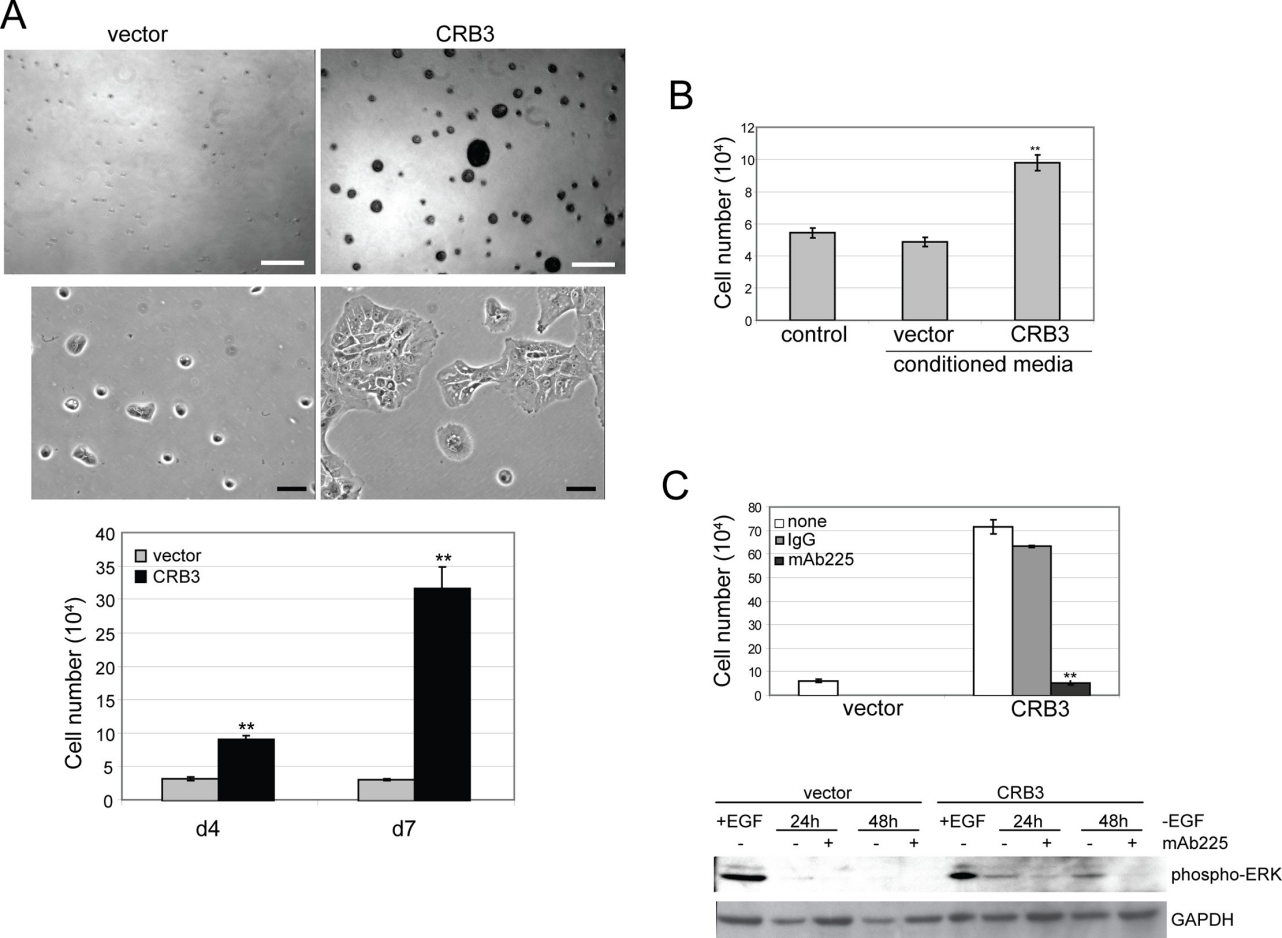
CRB3 expression leads to EGF-independent proliferation through a secreted factor. **(A)** Vector control or CRB3-expressing MCF-10A cells were cultured on Matrigel for 15 days (top) or in monolayer for seven days (middle) without EGF, and representative phase images of each are shown. Scale bars, 150 μm (3D), 50 μM (monolayer). EGF-independent proliferation in monolayer culture was quantified by cell counting (bottom). The results represent the mean +/-SEM from three independent experiments (n = 9) (**p<0.01 by Students *t* test). **(B)** Conditioned media was collected from vector control or CRB3-expressing cells following 5 days in culture without EGF. Proliferation of normal MCF-10A cells cultured in the conditioned media was quantified by cell counting on days 5–7 after addition of each conditioned medium. Results shown are average +/-SEM of four independent experiments with each condition in duplicate (**p<0.01 for CRB3 compared to vector using Students *t* test). **(C)** EGF-independent proliferation of CRB3 expressing cells is blocked by the addition of mAb225. Results are the average +/- SEM of three independent experiments with each condition in duplicate (n = 6) (**p<0.01 for mAb225 compared to IgG using Students *t* test) (top). CRB3 expressing cells were starved of EGF for the times indicated in the presence or absence of the EGFR blocking antibody, mAb225, lysed, and analyzed for phospho-ERK by SDS-PAGE and immunoblotting. Immunoblotting for GAPDH was used as a loading control (bottom).

MCF-10A cells are dependent on epidermal growth factor receptor (EGFR) signaling for proliferation under normal culture conditions. Because CRB3 expression induced a hyperproliferative phenotype in MCF-10A cells, we examined whether CRB3 expression would also support epidermal growth factor (EGF)-independent proliferation. Indeed, CRB3-expressing MCF-10A cells were able to sustain proliferation in the absence of EGF in both 3D basement membrane culture and in monolayer culture (Fig 2A). To ascertain whether this EGF-independent proliferation was autonomous or non-autonomous, we collected conditioned medium from EGF-independent monolayer cultures of CRB3-expressing cells and tested whether the medium could support the proliferation of normal MCF-10A cells. Unlike conditioned medium from normal or vector control cells, conditioned medium from CRB3-expressing cells was able to support EGF-independent proliferation of MCF-10A cells (Fig 2B). Similarly, normal MCF-10A cells marked with a green fluorescent protein derivative, eCit, were able to proliferate in the absence of EGF if they were co-cultured with CRB3-expressing MCF-10A cells (S1A Fig). These results demonstrated that increased proliferation in the CRB3-expressing structures was through a non-autonomous mechanism that involved a secreted factor.

Several lines of investigation provided further evidence that the ability of CRB3- expressing cells to proliferate in the absence of EGF is the result of increased EGFR signaling in response to increased secretion of the EGFR ligand, amphiregulin (AREG). First, we monitored activation of the EGFR pathway by immunoblot analysis in the CRB3-expressing cells to confirm activation of the pathway. In the absence of exogenous EGF, CRB3 cells maintained high levels of phospho-EGFR and phospho-ERK, compared to similarly treated control cells (S1B Fig). Increased EGFR signaling and EGF-independent proliferation were both blocked in the presence of a monoclonal antibody to EGFR, mAb225 (Fig 2C), suggesting that the secreted factor was an EGFR ligand.

**Fig 3:**
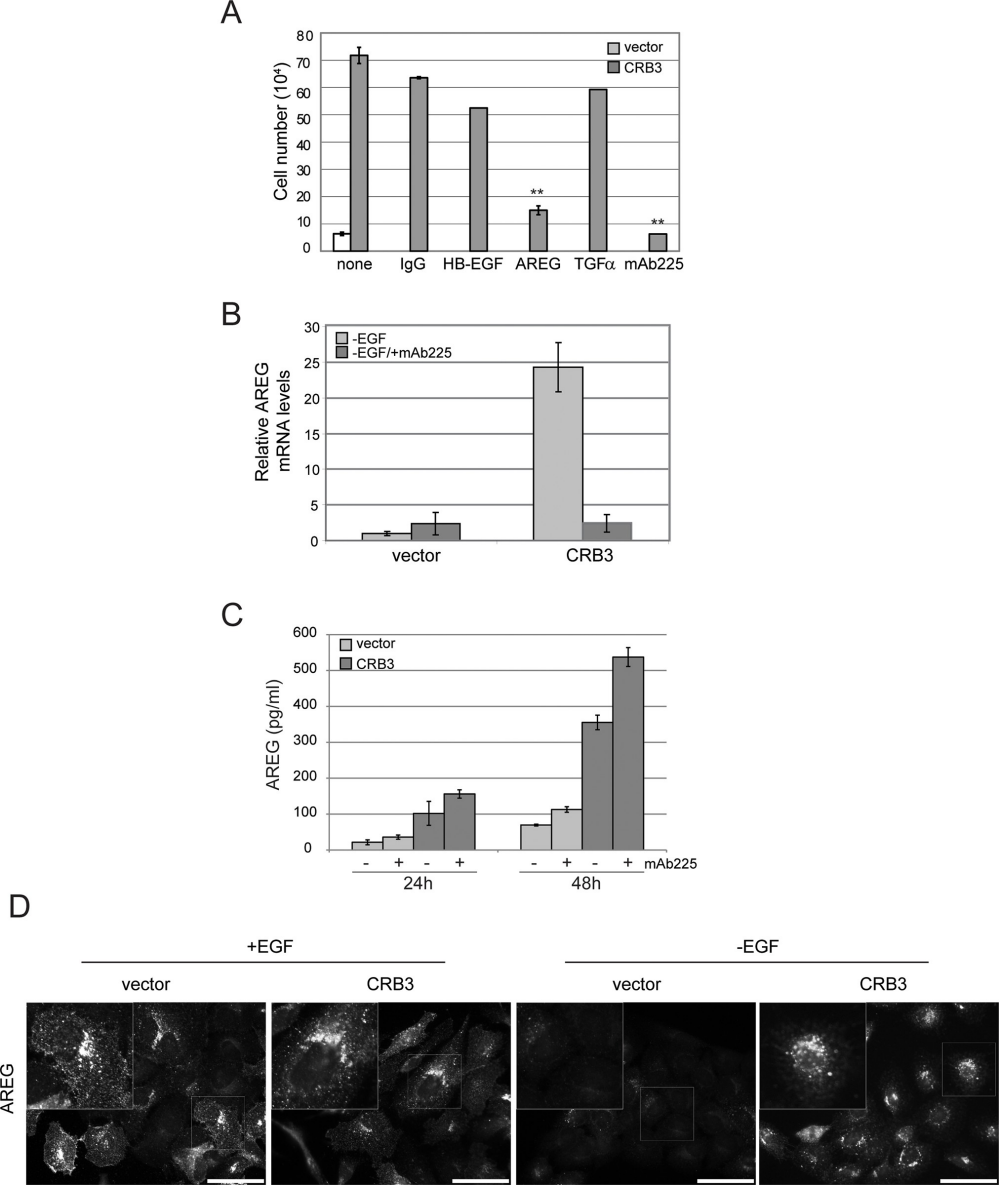
CRB3 expression leads to an increase in secreted amphiregulin. **(A)** The AREG neutralizing antibody blocks EGF-independent proliferation in CRB3 expressing cells. Results shown are the average +/- SEM of 2 independent experiments with each condition in triplicate (n = 6) (**p<0.01 for AREG and mAB225 compared to IgG using Students *t* test). **(B)** qRT-PCR showing that AREG mRNA levels are increased in CRB3-expressing cells, and the autocrine loop leading to increased AREG mRNA expression is blocked with mAb225. Results shown are the average +/-SEM of triplicate samples and are representative of three independent experiments. **(C)** AREG ELISA assay showing an increase in AREG protein levels in the conditioned media from CRB3-expressing MCF-10A cells cultured in the absence of EGF. AREG release is not blocked by treatment with mAb225. Results shown are the average +/- SEM of triplicate samples and are representative of three independent experiments. **(D)** Immunofluorescence with an AREG antibody shows AREG is localized to discrete puncta in MCF-10A cells, a signal which is lost upon EGF starvation but retained upon CRB3 expression in the absence of EGF. The number of AREG puncta is increased, and the puncta are more disperse in the CRB3-expressing cells. Scale bars, 50 μm.

In order to identify the secreted ligand, we used neutralizing antibodies to selectively block the activity of specific EGFR ligands in the EGF-independent proliferation assay and found that the neutralizing antibody targeting AREG led to a significant decrease in EGF-independent proliferation of CRB3-expressing cells (Fig 3A), while neutralizing antibodies against other EGFR ligands had no effect. AREG levels are regulated both at the transcriptional level through an EGFR-mediated autocrine loop and at the level of processing and secretion of the mature AREG ligand [13]. CRB3 expression led to an increase in *AREG* mRNA levels, suggesting that, as expected, increased AREG secretion sets up the well-described autocrine AREG loop which can support EGF-independent proliferation (S1C Fig). Indeed, the induction of the *AREG* mRNA transcript is EGFR-dependent, as it is blocked in the presence of mAb225 (Fig 3B). To distinguish between the possibility that the increase in AREG release was simply due to the increase in AREG expression through a transcriptional change in *AREG* mRNA levels from the more interesting possibility that the increase in AREG release was due to CRB3 acting directly at the level of AREG processing and secretion, we monitored AREG release in the presence of the EGFR neutralizing antibody (Fig 3C). Using a human AREG enzyme-linked immunosorbent assay (ELISA) assay to quantify the level of AREG secretion, we observed a more than 3-fold increase in AREG secretion from CRB3-expressing cells in the absence of exogenous EGF. Treatment of the CRB3-expressing MCF-10A cells with mAb225 to block EGFR signaling reduced *AREG* mRNA levels (Fig 3B); however, mAb225 did not reduce AREG release from CRB3-expressing cells (Fig 3C). These results demonstrate that CRB3 expression regulates AREG signaling at the level of processing or secretion of the active ligand, which in turn leads to increased *AREG* expression through a previously characterized autocrine loop involving EGFR. It is important to emphasize that despite the decrease in *AREG* mRNA levels and the block in ERK activity in the presence of mAb225, CRB3-expressing cells still secrete AREG into the media. Thus, the primary effect of CRB3 expression is to increase the processing and/or secretion of AREG, which has the secondary effect of establishing an autocrine loop to increase *AREG* mRNA levels.

We then monitored the changes in AREG levels in CRB3-expressing MCF-10A cells by immunofluorescence. In control cells, we observed a punctate distribution of AREG under standard culture conditions with EGF, and EGF starvation led to a reduction in AREG staining (Fig 3D). The loss of AREG staining following EGF starvation of control cells was consistent with the results of the AREG ELISA described above and reflects the expected drop in intracellular AREG levels following down regulation of the EGFR-dependent autocrine loop controlling AREG expression. Overexpression of CRB3 in MCF-10A cells increased the level of AREG staining under conditions of EGF-independent growth, consistent with the results of our AREG ELISA assay. Interestingly, not only were the number of AREG puncta increased but the localization of the AREG-containing puncta was significantly different than in vector control cells. Specifically, in the CRB3-expressing cells, the AREG puncta were more dispersed throughout the cytoplasm, in contrast to the tight perinuclear localization of the AREG puncta in vector control cells (Fig 3D). This dispersion of AREG puncta was also evident in the CRB3 cells under normal culture conditions in the presence of EGF, suggesting that the underlying change elicited by CRB3 expression was independent of AREG/EGFR signaling and the EGFR pathway and was a direct result of CRB3 expression.

### CRB3-dependent AREG secretion and EGF-independent proliferation utilize the FERM binding domain of CRB3

**Fig 4:**
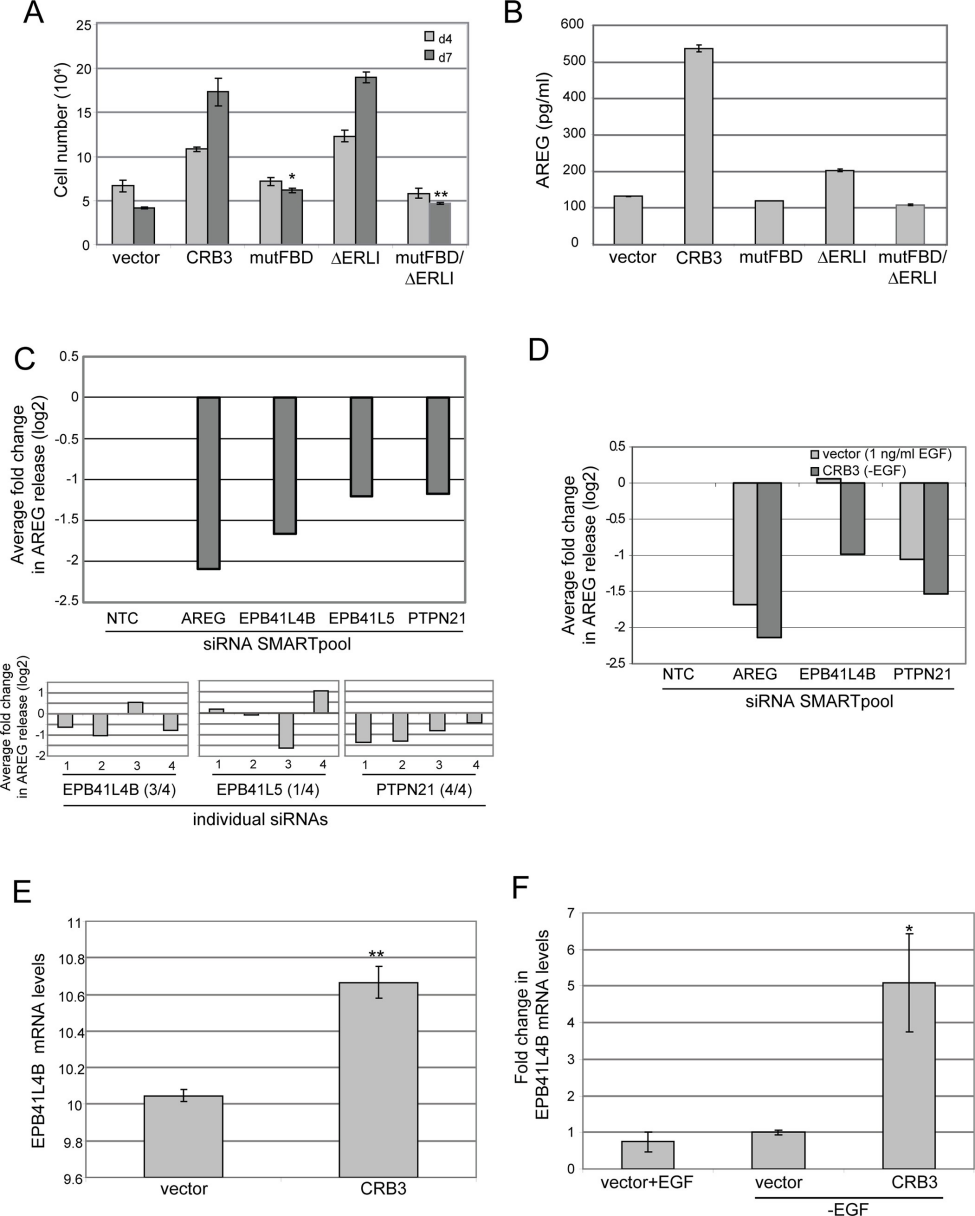
The FERM protein, EPB41L4B, is involved in AREG release from CRB3-expressing cells and is co-expressed with CRB3. **(A)** MCF-10A cells expressing the indicated CRB3 mutations were tested for EGF-independent proliferation and quantified by cell counting. Results shown are the average cell number +/- SEM of triplicate samples and are representative of three independent experiments (**p<0.01 using Students *t* test). **(B)** AREG levels in the conditioned media from cells expressing the indicated CRB3 mutants were measured using an AREG ELISA assay. Results shown are the average +/- SEM of triplicate samples and are representative of three independent experiments. **(C)** Hits from a screen of Dharmacon siRNA SMARTPools targeting genes with an annotated FERM domain whose knockdown give a 2-fold or greater inhibition of AREG release in CRB3-expressing MCF10A cells relative to a non-targeting control (NTC) siRNA pool. Values are reported on a log2 scale and are the average of duplicate samples from two independent screening experiments (top). Quantitation of the inhibition of AREG release using individual siRNA oligos targeting the indicated candidate FERM proteins. Parentheses indicate number of siRNAs with a log2 fold change of -0.5 or more (bottom). **(D)** Knockdown of EPB41L4B specifically inhibits AREG release from CRB3-expressing cells. Results shown are the average of duplicate samples. **(E)** Microarray analysis of MCF-10A cells expressing vector or CRB3 in the presence of EGF identified significantly increased expression of EPB41LB in CRB3 cells. Results are the average +/-SEM of triplicate samples (p = 3.45 x 10^−3^). **(F)** Validation of microarray data by qRT-PCR with mRNA levels shown as the fold change relative to vector -EGF. EPB41L4B expression does not change with addition of EGF in MCF-10A cells but is increased upon ectopic CRB3 expression, even in the absence of EGF (*p<0.05 using Students *t* test). Results shown are the average +/- SEM of triplicate samples and are representative of three independent experiments.

Knowing that CRB3 expression has a direct effect on the cellular machinery that is involved in processing or trafficking of AREG, we undertook domain analysis of the CRB3 intracellular region responsible for the effects of CRB3 on AREG release and EGF-independent proliferation. The CRB3 intracellular sequence contains two motifs that are conserved within *Drosophila* Crumbs and are identified in all mammalian *CRB* genes: a juxtamembrane FERM binding domain (FBD) and a carboxy-terminal PDZ binding domain with the sequence ERLI [4]. The PDZ domain has a well-established role in the CRB-dependent regulation of cell polarity, while the function of the FBD is not as well defined but is thought to link the apical CRB complex to the actin cytoskeleton [5]. The mammalian CRB3 isoform has an additional unique intracellular sequence (RxPPxP) with similarity to an SH3-domain binding site [4]. We stably expressed amino-terminal myc-tagged CRB3 variants with mutations in each of these domains [4] and tested the ability of these mutants to support EGF-independent proliferation (Fig 4A). Our results demonstrate that mutation of the PDZ binding domain (ΔERLI) did not affect the ability of CRB3 to support EGF-independent proliferation, indicating that the CRB3-dependent proliferative signal is distinct from the previously characterized role of the PDZ domain in regulating cell polarity. Similarly, mutation of the RxPPxP sequence (mutRP) in CRB3 did not impact EGF-independent proliferation. In contrast, expression of the CRB3 variant with a mutant FERM binding domain (mutFBD) at levels similar to the ΔERLI mutant (S2A Fig) did not induce EGF-independent proliferation. We then determined whether the loss in EGF-independent proliferation in the mutFBD cells was a consequence of a decrease in AREG secretion from these cells. While the ΔERLI-expressing cells were competent to secrete AREG, albeit at lower levels than wildtype cells, the mutFBD cells did not secrete increased levels of AREG (Fig 4B). Together, these findings define a previously uncharacterized role for the FBD domain of CRB3 in controlling the proliferation of mammary epithelial cells through the regulation of AREG secretion.

### Unbiased screens identify EPB41L4B as a FERM protein required for CRB3-induced AREG release and EGF-independent proliferation

Our data demonstrate that overexpression of the polarity protein CRB3 elicits changes in the MCF-10A cells that culminate in an increase in the release of AREG and the subsequent activation of EGFR signaling to drive proliferation. The events downstream of CRB3 leading to AREG release require the FBD of CRB3. To identify candidate FERM proteins responsible for the CRB3-mediated proliferation and AREG release, we took an unbiased approach using two complementary experimental strategies. First, we carried out a focused siRNA screen on a collection of genes from the human genome with annotated FERM domains to identify the proximal CRB3 binding partner required for the increase in AREG release mediated by the CRB3 FBD. In a second, parallel line of investigation, we set out to define global changes in the transcriptional landscape induced by CRB3 expression in mammary epithelial cells using microarray analysis on the MCF-10A cells with ectopic CRB3. Remarkably, these approaches converged on the same gene, EPB41L4B, and we show here that EPB41L4B plays an essential role in mediating the proliferative signal downstream of CRB3.

The RNA interference screen was carried out using a focused panel of SMARTpool siRNAs (Dharmacon) targeting 27 out of the 46 annotated human FERM proteins. This panel was assembled to focus on FERM proteins with established roles in kinase-mediated signal transduction and cytoskeletal regulation (S1 File), with the goal of identifying FERM proteins whose silencing prevents AREG release upon EGF deprivation of CRB3-expressing cells. This screen also included siRNAs to known PDZ binding partners of CRB3 (PARD6A, B, and G; MPP5/PALS1), to further address whether the PDZ binding domain is required for CRB3-dependent AREG release.

Using a human AREG ELISA assay to test cells transfected with this panel of siRNAs, we identified three FERM family genes whose silencing led to a decrease in AREG release in CRB3-expressing cells (Fig 4C). To validate these hits, we used four individual siRNAs against each gene and identified two of the genes, *EPB41L4B* and *PTPN21*, as validated components of the CRB3 pathway based on the criteria that at least two independent siRNAs resulted in a significant inhibition of AREG release. We then examined whether silencing of these genes was specific to the EGF-independent, CRB3-dependent AREG release or whether they were also required for the EGF-driven AREG release in normal MCF-10A cells. Loss of *PTPN21* inhibited AREG release in both contexts, but loss of *EPB41L4B* only decreased AREG release in the CRB3-expressing cells (Fig 4D), indicating that EPB41L4B functions specifically downstream of CRB3 to regulate AREG release. We also confirmed that the PDZ binding partners tested did not significantly affect AREG release in the CRB3 cells (S2B Fig), providing further evidence that known PDZ-dependent signals to the polarity machinery are not required for the CRB3-dependent pathway regulating AREG secretion.

For the microarray analysis, we isolated mRNA from CRB3-expressing MCF-10A cells and compared the expression profile to mRNA isolated from vector control cells. Examination of the microarray data showed significant changes in the transcript levels of a relatively small subset of genes in response to CRB3 expression. In particular, gene set enrichment analysis showed a statistically significant enrichment for genes involved in cell junction formation and epithelial maintenance and reduction of expression in genes associated with epithelial-to-mesenchymal transition among the genes that showed a greater than 1.5-fold change in expression in response to CRB3 expression (S2C Fig).

In response to ectopic CRB3 expression in MCF-10A cells, the expression of the FERM family protein EPB41L4B increased significantly (1.54-fold change, p = 3.45 x 10^−3^) (Fig 4E). To validate our microarray findings and to determine if EPB1L4B expression was induced as a secondary response to upregulated EGFR signaling or more directly in response to CRB3 overexpression, we used qRT-PCR analysis to monitor EPB1L4B transcript levels. First, we confirmed that the levels of EPB41L4B mRNA increased upon ectopic CRB3 expression as compared to vector control cells (4-fold change, p = 0.033). These changes in EPB41L4B expression were not affected by changes in EGFR signaling, as treatment of vector control cells with EGF did not affect EPB41L4B transcript levels (Fig 4F).

To address the general significance of this finding, we examined whether the relationship between CRB3 and EPB41L4B expression is observed more broadly in immortalized breast epithelial cell lines and in tumor cell lines derived from patient samples. Using published microarray studies, we analyzed both EPB41L4B and its close homologue, EPB41L5 (these two proteins are 62% identical and in *Drosophila* are represented by a single evolutionarily conserved homologue, Yurt [14]). Interestingly, we identified statistically significant co-expression of CRB3 with EPB41L4B and/or EPB41L5 in several microarray studies of cell line panels [15–17], across the entire cell line panel of the Cancer Cell Line Encyclopedia [18], as well as a study of 21 primary human mammary epithelial cell lines [19] (Table 1). Based on data generated by the TCGA Research network http://cancergenome.nih.gov/, there is a statistically significant positive correlation between CRB3 and EPB41L4B expression (r = 0.30, p = 4.1 x 10^−6^) in estrogen receptor (ER) negative TCGA breast samples. Although there is no significant correlation across the whole breast set or in ER positive tumors (r = 0.08 and r = 0.02, respectively), the two genes are generally co-expressed across the breast set, as they are both high expressed in the vast majority of breast samples. Taken together, these data suggest that co-expression of CRB3 and EPB41L4B is a general phenomenon.

**Table 1:** Co-expression of CRB3 and the FERM proteins EPB41L4B and EPB41L5.

Gene	r-value	p-value	Study (Reference)
EPB41L4B	0.84	2.2 x 10^−6^	Ince (16)
	0.69	2.5 x 10^−18^	Wagner (12)
	0.61	1.1 x 10^−22^	Adai (13)
	0.58	4.0 x 10^−88^	Barrentina (15)
	0.47	4.5 x 10^−4^	Hoeflich (14)
EPB41L5	0.84	1.7 x 10^−6^	Ince (16)
	0.75	5.8 x 10^−23^	Wagner (12)
	0.71	3.8 x 10^−9^	Hoeflich (14)
	0.70	1.3 x 10^−31^	Adai (13)
	0.39	8.3 x 10^−36^	Barrentina (15)

Table of the Pearson coefficients (r-values) and p-values from correlation analysis of *CRB3* and *EPB41L4B* or *EPB41L5* mRNA in the listed studies. The r-value is a measure of the linear association between two variables between -1 and 1; positive r-values indicate a positive linear relationship. The threshold for statistically significant correlation was p = 0.05.

**Fig 5:**
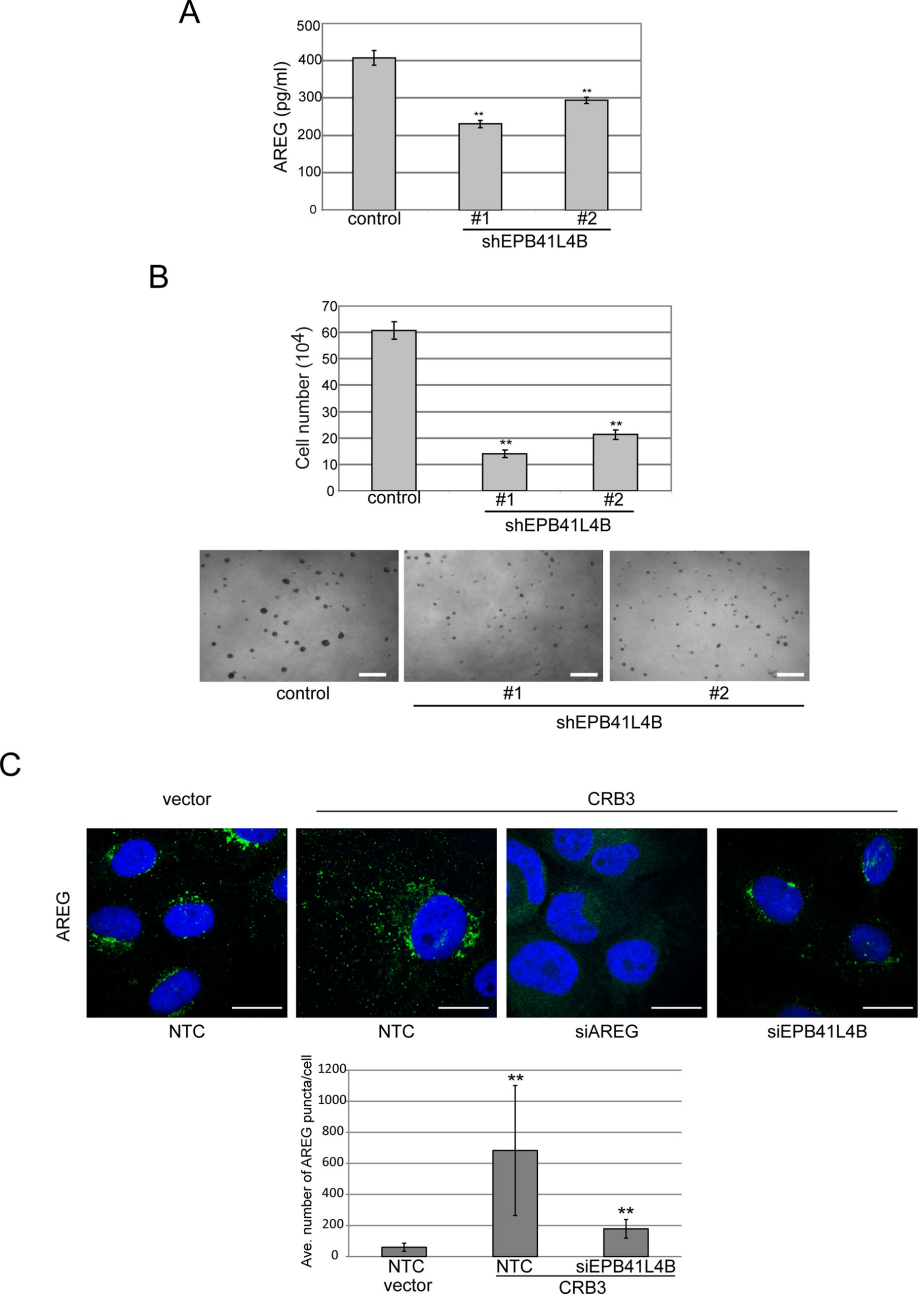
The FERM protein EPB41L4B is required for EGF-independent proliferation and AREG secretion in CRB3-expressing cells. **(A)** Stable knockdown of EPB41L4B expression suppresses AREG release from CRB3-expressing cells measured by the AREG ELISA assay. Results shown are the average +/-SEM of triplicate samples and are representative of three independent experiments (**p<0.01 using Students *t* test) (left). **(B)** EGF-independent proliferation in CRB3 cells was blocked with EPB41L4B shRNA in both monolayer (top) and 3D cultures (bottom). Cell proliferation for monolayer culture was quantified by cell counting on day 7, and the results shown are the average +/- SEM of triplicate samples and are representative of three independent experiments (**p<0.01 using Students *t* test). Representative phase images for EGF-independent 3D culture are shown. Scale bars, 150 μm. **(C)** Loss of EPB41L4B reverts the dispersion of AREG-positive puncta in the CRB3 expressing cells. AREG localization and distribution in control cells or CRB3-expressing cells with the indicated siRNA treatments was imaged by confocal to quantify the average number of AREG-positive puncta per cell. Error bars shown are +/-SD for the average number of AREG-positive puncta per cell from counts on 20–25 cells per condition and are representative of three independent experiments (**p<0.01 using Students *t* test). Scale bars, 10 μm.

To examine whether EPB41L4B was required for the CRB3-dependent, EGF-independent proliferation, we created stable short hairpin RNA lines for EPB41L4B in the background of CRB3 overexpression and validated the knockdown of EPB41L4B with qRT-PCR (S3A Fig). Using the AREG ELISA assay, we confirmed that stable knockdown of EPB41L4B led to a decrease in AREG release from CRB3-expressing cells (Fig 5A). As shown in Fig 5B, EPB41L4B knockdown caused a loss of EGF-independent proliferation in both monolayer and basement membrane culture. Silencing of EPB41L4B using siRNA (S3B Fig) also reverted the changes in AREG levels and localization of puncta observed by immunofluorescence (Fig 5C). Specifically, CRB3-expressing cells transfected with a control siRNA showed significantly more AREG-containing puncta dispersed throughout the cytoplasm compared to vector control cells. Knockdown of EPB41L4B resulted in a decrease in AREG puncta, most notably as a restriction of the AREG puncta to a tight perinuclear region, similar to the localization of AREG in vector control MCF-10A cells (Fig 5C). Quantification of the reduction in AREG-positive puncta revealed that the number of AREG-positive puncta increased approximately 10-fold upon CRB3 expression and that silencing of EPB41L4B reverted the number of AREG-positive puncta numbers close to vector control. The reduction in the number of AREG-positive puncta following EPB41L4B knockdown is in contrast to the complete loss of AREG-staining in cells treated with siRNA against *AREG*, which completely abrogates AREG-positive puncta, providing confirmation that the puncta detected with the AREG antibody indeed AREG. Thus, these findings provided evidence that EPB41L4B plays a critical role in the signaling events downstream of CRB3 that lead to AREG release and the ability of these cells to proliferate in the absence of exogenous growth signals.

Taken together, our microarray and siRNA data identified EPB41L4B as a FERM family protein that is upregulated at the transcriptional level in response to ectopic expression of CRB3 and is required for CRB3-induced AREG release and proliferation. Given the requirement of the FBD of CRB3 for mediating the proliferative phenotype, this data supports a model in which EPB41L4B interacts with CRB3 to mediate the cellular changes responsible for AREG release.

### CRB3 signaling through EPB41L4B alters the size and distribution of endocytic vesicles

**Fig 6:**
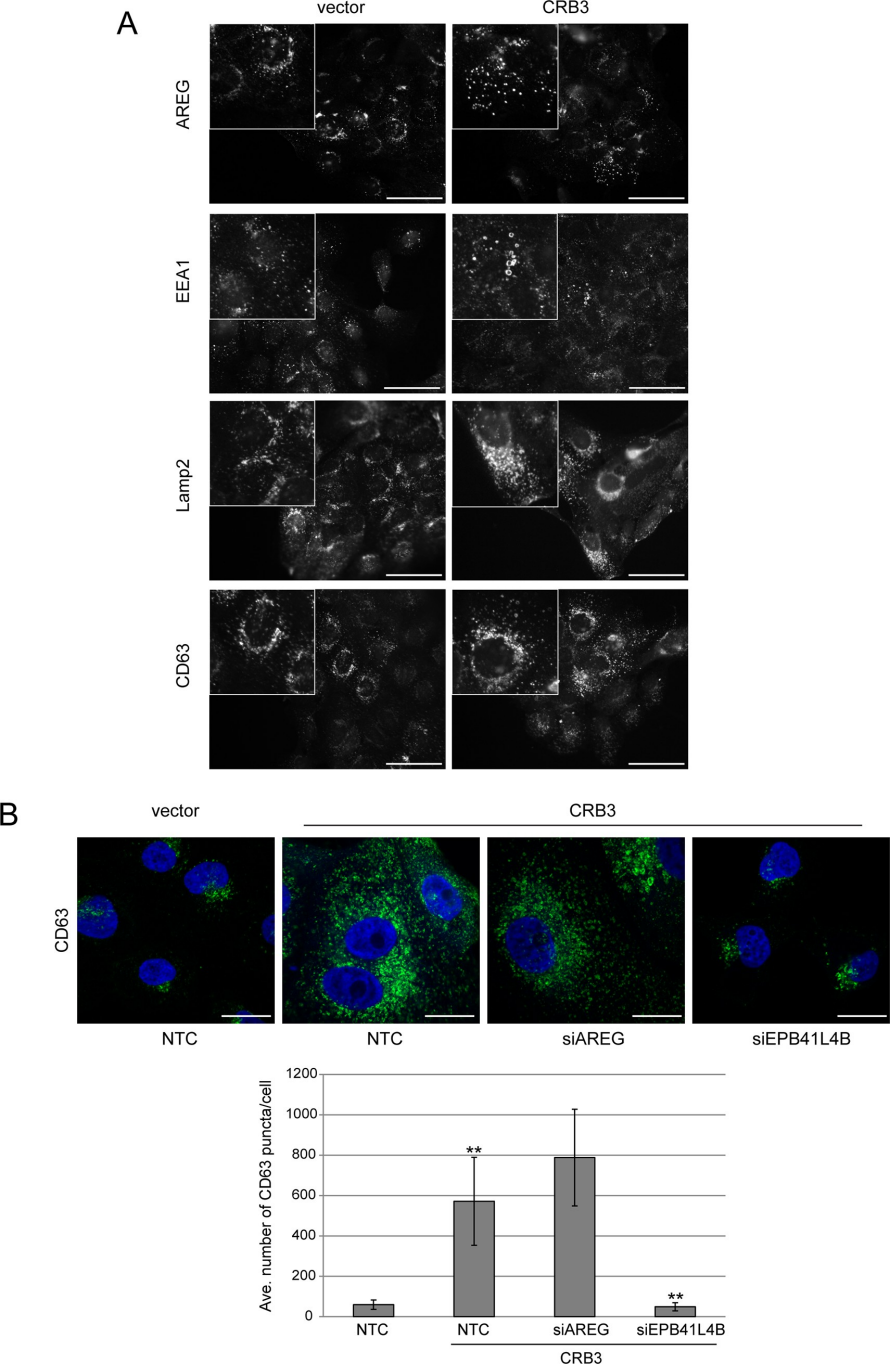
CRB3 expression leads to an increase in the number and size of CD63-positive puncta, and loss of EPB41L4B in the CRB3 background reverts this phenotype. **(A)** Confocal imaging of endocytic markers shows that CRB3 expression results in an increase in the number and size of endocytic vesicles, including those of early (EEA1) and late (LAMP-2, CD63) endosomes. Scale bars, 50 μm. Insets provide a higher magnification view of the same field to highlight vesicular structures. **(B)** Loss of EPB41L4B (but not AREG) reverts the dispersion of CD63-positive puncta in the CRB3 expressing cells. Control cells or CRB3-expressing cells with the indicated siRNA treatments were imaged by confocal (top) to quantify the average number of CD63-positive puncta per cell (bottom). The number of CD63-postive puncta increases approximately 10-fold upon CRB3 expression and is reverted to numbers close to vector control by loss of EPB41L4B. Error bars shown are +/-SD for the average number of CD63-positive puncta per cell from counts on 20–25 cells per condition and are representative of three independent experiments (**p<0.01 using Students *t* test). Scale bars, 10 μm.

CRB3 expression led to an EPB41L4B-dependent increase in the number of AREG puncta and changes in their cytoplasmic distribution. To further characterize the changes in AREG trafficking regulated by CRB3 and EPB41L4B, we immunostained CRB3-expressing cells with the early endosome marker EEA1 and the late endosome/lysosome markers LAMP-2 and CD63. We found that CRB3 expression led to a dramatic increase in the size of early endosomes marked with EEA1, as well as an increase in the size and dispersion of both LAMP-2- and CD63-positive vesicles (Fig 6A). Because the most visible changes were in vesicles marked by CD63, we further characterized the CRB3-dependent changes and the role of EPB41L4B in those changes using this marker. Using confocal images of CRB3-expressing MCF-10A cells transfected with siRNAs against AREG and EPB41L4B, we found that the number of CD63-postive puncta increased approximately 10-fold upon CRB3 expression and silencing of EPB41L4B reverted the numbers to values similar to vector control (Fig 6B). Knockdown of AREG by siRNA did not affect the formation and localization of the CD63-marked vesicles in the CRB3 cells, providing additional evidence that the changes in endocytic vesicles are not dependent on the EGFR signaling elicited by CRB3 and are a direct effect of CRB3 on the vesicular trafficking machinery.

### Expression of EPB41L4B enhances CRB3-dependent AREG secretion and formation of endocytic vesicles

**Fig 7:**
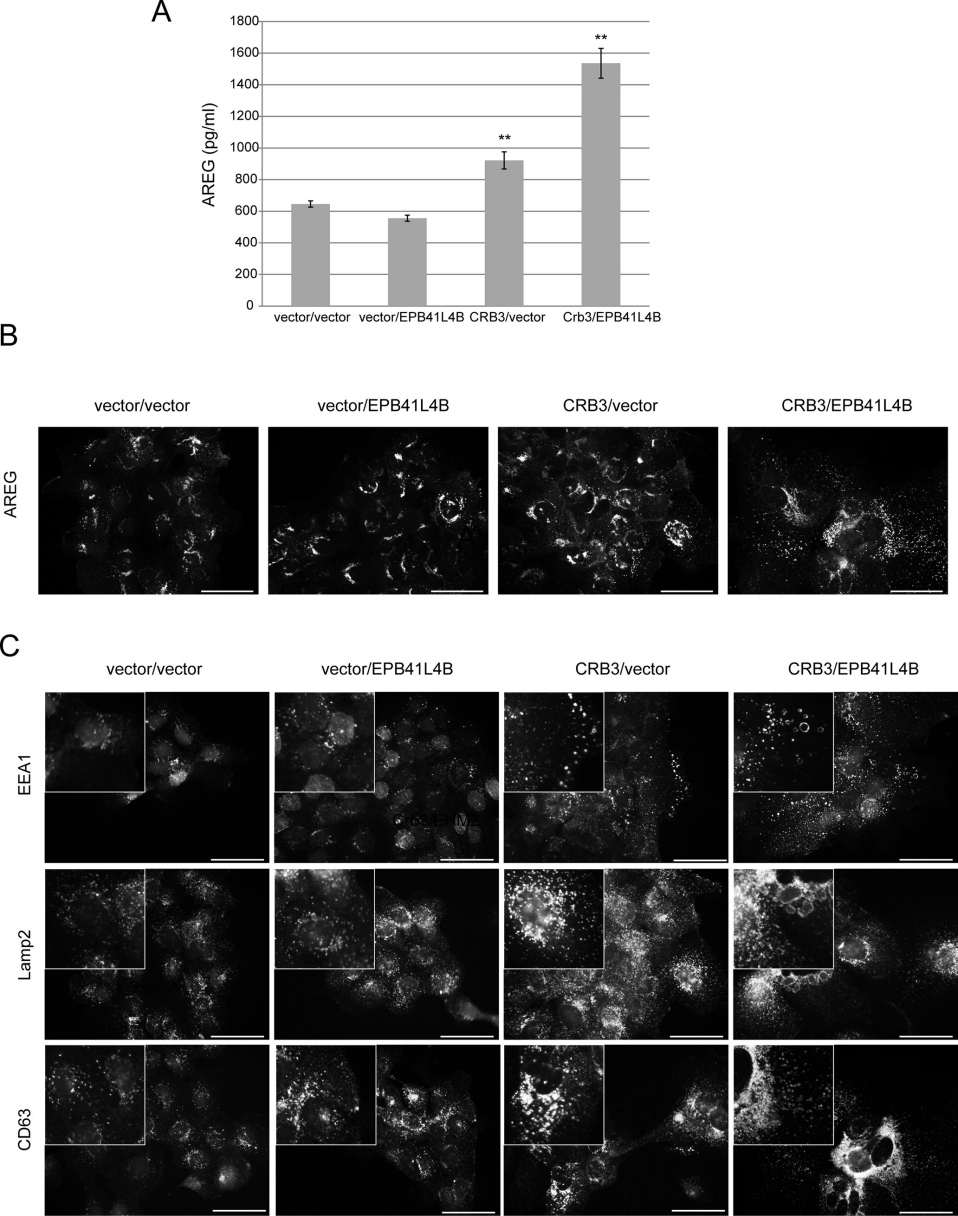
Expression of EPB41L4B enhances CRB3-dependent AREG secretion and formation of endocytic vesicles. **(A)** Co-expression of EPB41L4B enhances AREG levels in the medium from CRB3-expressing cells as measured by the AREG ELISA. Results shown are the average +/- SEM of 2 independent experiments with each condition in triplicate (n = 6) (**p<0.01 for CRB3/vector compared to vector/vector and for CRB3/EPB41L4B compared to CRB3/vector using Students *t* test). **(B)** Co-expression of EPB41L4B increases the dispersion of AREG-positive puncta in CRB3-expressing cells. **(C)** Co-expression of EPB41L4B enhances the number and size of endocytic vesicles, including those of early (EEA1) and late (LAMP-2, CD63) endosomes in CRB3-expressing cells. Scale bars, 50 μm. Insets provide a higher magnification view of the same field to highlight vesicular structures.

Our conclusions were further supported by studies on MCF-10A cells co-expressing CRB3 and EPB41L4B. We expressed an HA-tagged variant of EPB41L4B alone or in combination with myc-CRB3 (S4A Fig) and found that while EPB41L4B alone did not support AREG secretion in the absence of EGF, co-expression of EPB41L4B with CRB3 resulted in an increase in AREG release above levels induced by CRB3 expression alone (Fig 7A). Furthermore, expression of EPB41L4B alone did not alter the immunostaining pattern of AREG, while the combination of CRB3 and EPB41L4B together caused a more dramatic change in the number and distribution of AREG puncta than those observed in CRB3-expressing cells (Fig 7B). The role of EPB41L4B in CRB3-dependent changes in vesicular trafficking was further confirmed upon examination of cells co-expressing CRB3 and EPB41L4B. Expression of EPB41L4B alone did not alter the number or distribution of endocytic markers. However, the expression of EPB41L4B in the CRB3 cells led to a more dramatic change in the number and distribution of endocytic markers, particularly LAMP-2 and CD63, the markers of late endosomes/lysosomes (Fig 7C). Interestingly, co-expression of CRB3 and EPB41L4B led to large vacuolar structures visible under low magnification phase contrast microscopy (S4B Fig).

## Discussion

We investigated the role of the polarity protein CRB3 in cell polarity and proliferation using the MCF-10A 3D culture model of mammary morphogenesis, an *in vitro* model that retains features of the organization of glandular mammary structures in basement membrane culture. We found that ectopic expression of CRB3 in MCF-10A cells induced a hyperproliferative phenotype in acini cultured in reconstituted basement membrane protein gels, as well as EGF-independent proliferation in both 3D and monolayer cultures. These CRB3-induced changes in proliferation were caused by increased release of AREG and mediated by the FBD of CRB3. We identified the FERM protein, EPB41L4B, as an essential mediator of CRB3-driven proliferation. In parallel, we observed CRB3-dependent changes in endocytic trafficking, a phenotype that was also dependent on EPB41L4B. Taken together, our data reveal a previously uncharacterized role for CRB3 in regulating proliferation in mammalian cells.

Our data demonstrate that the CRB3 proliferative phenotypes are driven by changes in AREG secretion, rather than direct changes in *AREG* transcription. This subsequently leads to increased AREG expression through a previously characterized AREG-dependent positive feedback loop. The changes in endocytic markers we observed following CRB3 expression are independent of EGF signaling, and knockdown of *AREG* does not alter the CRB3-dependent changes in the endocytic machinery. That we observe endocytic changes independently of AREG signaling argues strongly that the proliferative signal caused by CRB3 arises directly from defects in endocytic trafficking that lead to an increase in AREG release and inappropriate signaling through the EGF-receptor pathway. These results support a model in which changes in endocytic trafficking are the fundamental defect caused by CRB3 overexpression.

The proliferative phenotypes induced by CRB3 overexpression in mammary cells reported here provide evidence in mammalian cells for a direct connection between junctional complexes, endocytosis, and proliferation. Our findings are supported by a number of important genetic studies in flies that first established a connection between cell proliferation and junctional complexes [20, 21]. Specifically, screens for mutations that produced tumor-like growths in flies showed that loss of junctional scaffold proteins, such as *lethal giant larvae (lgl)* and *discs large (Dlg)*, induced a hyperproliferative phenotype in imaginal discs and follicular epithelia [22]. Mutations in these tumor suppressor genes resulted in aberrant cell growth accompanied by a distinct alteration of polarity, namely expansion of the apical membrane, a phenotype also observed upon overexpression of certain apical membrane determinants, such as *crumbs (Crb)*. In flies, loss of *avl*, a syntaxin localized to early endosomes, or Rab5, a regulator of trafficking to early endosomes, not only caused an expansion of the apical membrane but also resulted in overproliferation and accumulation of the Notch receptor and Crb, due to endocytic failure. Ectopic expression of the *Drosophila* Crb protein similarly induced apicalization of the membrane and hyperproliferation of imaginal discs, a phenotype that was nearly identical to that induced by endocytic failure in *avl* mutants [23]. Interestingly, mutants in the ESCRT pathway required for endosomal trafficking showed increased levels of Crb that was mislocalized into aberrant endosomal structures, as well as overproliferation of imaginal discs [24]. Furthermore, mutations in the *Drosophila* ortholog of the mammalian TSG101 and consequent defects in endocytosis, led to non-autonomous proliferation through secretion of a growth factor, similar to the CRB3-induced proliferative phenotype that is dependent on release of and signaling through AREG, a ligand for the EGF receptor. The phenotypic similarities between the fly mutants and our CRB3 overexpression data reported here provide strong support for a model in which junctional scaffolding proteins regulate cell proliferation through effects on endocytic trafficking.

We sought to define proximal partners for CRB3 in these processes and found that in monolayer culture, AREG secretion was dependent on the FBD of CRB3, determined by functional domain analysis of CRB3. Importantly, the PDZ binding domain is not required for the EGF-independent proliferation, a finding confirmed through siRNA of known PDZ binding partners, including PALS1 and PAR6. Our data implicating the FBD of CRB3 in proliferative signaling is significant, because it argues strongly that the mechanism of proliferative signaling involves a pathway distinct from the well-characterized, PDZ-dependent function of CRB3 in establishing polarity in mammalian cells. Specifically, the PDZ domain of CRB3 has been implicated in regulation of polarity through a mechanism that involves signaling through atypical PKC isoforms, as described above. Thus, our data identifies an important new function of the FBD of CRB3 in regulating cell proliferation in mammalian cells.

By screening a large subset of the FERM-containing proteins in the human genome using siRNA technology, we identified the FERM protein EPB41L4B (also known as EPB41L4B/Lulu2) as having an important role in the CRB3-dependent AREG secretion and EGF-independent proliferation. We also identified this gene as an upregulated gene in a microarray study of MCF-10A cells overexpressing CRB3. Furthermore, we found EPB41L4B to be required for the CRB3-dependent changes in endocytosis, particularly on the increase in CD63-positive vesicles. Interestingly, the orthologs for this FERM protein in *Drosophila* and zebrafish, Yurt and mosaic eyes (Moe), respectively, have been shown to negatively regulate Crb-dependent apical membrane formation in developing epithelia, such that Yurt mutants led to expansion of the apical membrane [14, 25]. In this same work, the mammalian orthologs of Yurt, EPB41L4B (EPB41L4B) and EPB41L5 (Ymo1), could each interact with all three mammalian isoforms of Crumbs, Crb1, Crb2, and CRB3. Our findings extend this data on the biochemical interaction to show that the CRB3/EPB41L4B interaction plays a functional role in regulation of endocytic trafficking and proliferation. In flies, defects in polarity caused by Yurt mutants were confined to head morphogenesis and dorsal closure, and many of the epithelial polarity defects were recovered by the end of embryogenesis. The FBD of CRB3 was not required for tight junction formation in MCF-10A cells [4], and we show here that EPB41L4B appears to be the critical FERM protein connecting CRB3 to proliferative signaling in mammary epithelia. Studies in MDCK cells on EPB41L4B and EPB41L5 showed that EPB41L4B isoforms, when overexpressed, increased the apical staining of CRB3 [26]. Our results here show an enhanced proliferative and endocytic phenotype when CRB3 and EPB41L4B are coexpressed. As noted above, *Drosophila* Yurt has two orthologs in mammals, EPB41L4B and EPB41L5, which both contain a FERM domain and FA domain with 62% protein identity. It is not yet clear whether these two orthologs have distinct or complementary roles in mammalian cells, or whether the expression of EPB41L4B and EPB41L5 may be regulated developmentally, such that these proteins play distinct roles in regulation of polarity and proliferation at different stages of development. Our transcriptional data shows strong correlation between both EPB41L4B and EPB41L5 expression and expression of CRB3 in various cell lines, suggesting that at least one of these FERM proteins is required for CRB3 functionality in epithelia.

Little is known about the specific functions of mammalian EPB41L4B. Given that FERM proteins in general regulate the actin cytoskeleton, it is a simple hypothesis to suggest that CRB3/EPB41L4B modulates the actin cytoskeleton to impact endocytosis. Indeed, recent data demonstrate that the interaction of CRB3A with EPB41L4B controls epithelial cell morphology and cell cohesion through changes in actin and myosin II organization following recruitment and activation of p114RhoGEF and subsequent Rho signaling pathways [26–28]. EPB41L4B was first reported as a gene highly expressed in metastatic melanoma cell lines [29] and is reported to be overexpressed in prostate cancer [30]. Loss of EPB41L4B led to in inhibition of *in vitro* proliferation of breast cancer cell lines, and increased EPB41L4B levels were correlated with disease progression and poor prognosis [31].

Studies in *Drosophila* have indicated that the FBD of Crb may interact with the Expanded protein [32–35], a FERM domain protein that regulates the function of Hippo pathway in growth control [36]. Overexpression of Crb in *Drosophila* led to loss of Expanded and activation of the Hippo-Warts-Yorkie pathway, leading to cell overgrowth. As noted above, our data implicate trafficking alterations leading to increased AREG release as a primary driver of the proliferative phenotype induced by CRB3 expression; however, it is likely that there are additional layers of regulation in this process. Crosstalk between the Hippo pathway and the Crb complex remains largely unexplored in mammalian cells. It is interesting to note that both Lgl and Crb contribute to crosstalk with the Hippo pathway in *Drosophila* [34] and that the Hippo pathway has been shown to regulate apical membrane formation independently of its growth control function, further linking these two pathways. Given that both Hpo and Wts mutants led to accumulation of the endosomal sorting protein, Hrs, it is intriguing to hypothesize that endocytosis may be the common link between the various aspects of crosstalk between the Hippo pathway and the polarity regulators.

## Supporting information

S1 Fig CRB3 expression leads to EGF-independent proliferation through a secreted EGFR ligand. **(A)** Like vector control cells, the eCit expressing cells are unable to proliferate in the absence of EGF (top). Co-culture of eCit labeled MCF-10A cells with CRB3 expressing cells allows the eCit cells to proliferate in the absence of EGF. Representative phase and fluorescent images of the co-culture are shown (middle). The cell number of MCF-10A eCit cells was quantified after 5 days of culture in conditioned medium from the indicated cell types (bottom). **(B)** Western blot showing phospho-EGFR and phospho-ERK levels are elevated in CRB3 cells in the absence of EGF. **(C)** qRT-PCR reveals that CRB3 expression maintains *AREG* expression in the absence of EGF.
(EPS)

S2 Fig An siRNA-based screen of FERM proteins identified EPB41L4B as a specific regulator of CRB3-dependent phenotypes in MCF-10A cells. **A)** Myc-tagged CRB3 mutants are expressed to equal levels in MCF-10A cells. 50 μg of total cell lysates were analyzed by immunoblotting with a polyclonal CRB3 antibody (raised against the carboxy-terminus of CRB3) (top) or a myc-epitope antibody, 9E10, against the amino-termical myc-tag (bottom), with β-tubulin as a loading control. **(B)** Knockdown of gene expression for known binding partners to the PDZ binding domain of CRB3 does not interfere with AREG release in the CRB3-expressing cells. All siRNAs, except PARD6G, fell below the 1-fold inhibition threshold for AREG release. PARD6G did not validate with the individual siRNA oligos from the SMARTpool upgrade. **(C)** Microarray enrichment analysis of differentially expressed genes in CRB3-expresssing MCF-10A cells. Bars represent enrichment scores, defined as -log(pValue), of the top pathways identified by GeneGO enrichment analysis (MetaCore, GenGO; Thomson Reuters). The dashed line designates the threshold for statistical significance (p = 0.05).
(EPS)

S3 Fig Validation of EPB41LB silencing in CRB3-expressing MCF-10A cells. **(A)** Stable expression of EPB41L4B shRNAs and (**B**) an EPB41L4B SMARTpool decrease the expression of EPB41L4B in CRB3-expressing cells as measured by qRT-PCR. Results shown are the average +/- SEM of triplicate samples and are representative of three independent experiments (**p<0.01 using Students *t* test).
(EPS)

S4 Fig Co-expression of an HA-tagged EPB41L4B murine ortholog with CRB3 in MCF-10A cells. **(A)** The murine ortholog of EPB41L4B was expressed to equal levels in vector control and CRB3-expressing MCF-10A cells. 50 μg of total cell lysate was analyzed by immunoblotting with an HA-epitope antibody, 6E2, (top) and a polyclonal antibody against CRB3 (bottom) with GAPDH as a loading control. **(B)** Representative phase images of MCF-10A cells expressing CRB3 alone or co-expressing CRB3 and EPB41L4B. Scale bars, 50 μm.
(EPS)

S1 File Primary data from siRNA screen of FERM proteins. Genes with an annotated FERM domain were analyzed for reduced AREG secretion in CRB3-expressing MCF-10A cells using the AREG ELISA. Data are the calculated AREG amounts in pg/mL. Data are representative of n = 3 experiments.
(XLSX)

## References

[1] Huang L, Muthuswamy SK. Polarity protein alterations in carcinoma: a focus on emerging roles for polarity regulators. Curr Opin Genet Dev. 2010;20(1):41–50. Epub 2010/01/23. doi: S0959-437X(09)00198-1 [pii] 10.1016/j.gde.2009.12.001 ; PubMed Central PMCID: PMC3015045. 20093003 10.1016/j.gde.2009.12.001 PMC3015045

[2] Debnath J, Mills KR, Collins NL, Reginato MJ, Muthuswamy SK, Brugge JS. The role of apoptosis in creating and maintaining luminal space within normal and oncogene-expressing mammary acini. Cell. 2002;111(1):29–40. Epub 2002/10/10. doi: S0092867402010012 [pii]. . 12372298 10.1016/s0092-8674(02)01001-2

[3] Muthuswamy SK, Li D, Lelievre S, Bissell MJ, Brugge JS. ErbB2, but not ErbB1, reinitiates proliferation and induces luminal repopulation in epithelial acini. Nat Cell Biol. 2001;3(9):785–92. Epub 2001/09/05. 10.1038/ncb0901-785 [pii]. ; PubMed Central PMCID: PMC2952547. 11533657 10.1038/ncb0901-785 PMC2952547

[4] Fogg VC, Liu CJ, Margolis B. Multiple regions of Crumbs3 are required for tight junction formation in MCF10A cells. J Cell Sci. 2005;118(Pt 13):2859–69. Epub 2005/06/25. doi: 118/13/2859 [pii] 10.1242/jcs.02412 . 15976445 10.1242/jcs.02412

[5] Margolis B. The Crumbs3 Polarity Protein. Cold Spring Harbor perspectives in biology. 2018;10(3). Epub 2017/03/16. 10.1101/cshperspect.a027961 . 28289060 10.1101/cshperspect.a027961 PMC5830892

[6] Li P, Wang Y, Mao X, Jiang Y, Liu J, Li J, et al CRB3 downregulation confers breast cancer stem cell traits through TAZ/beta-catenin. Oncogenesis. 2017;6(4):e322 Epub 2017/04/25. 10.1038/oncsis.2017.24 ; PubMed Central PMCID: PMCPMC5520500. 28436991 10.1038/oncsis.2017.24 PMC5520500

[7] Li P, Feng C, Chen H, Jiang Y, Cao F, Liu J, et al Elevated CRB3 expression suppresses breast cancer stemness by inhibiting beta-catenin signalling to restore tamoxifen sensitivity. Journal of cellular and molecular medicine. 2018;22(7):3423–33. Epub 2018/03/31. 10.1111/jcmm.13619 ; PubMed Central PMCID: PMCPMC6010813. 29602199 10.1111/jcmm.13619 PMC6010813

[8] Debnath J, Muthuswamy SK, Brugge JS. Morphogenesis and oncogenesis of MCF-10A mammary epithelial acini grown in three-dimensional basement membrane cultures. Methods. 2003;30(3):256–68. Epub 2003/06/12. doi: S104620230300032X [pii]. . 12798140 10.1016/s1046-2023(03)00032-x

[9] Makarova O, Roh MH, Liu CJ, Laurinec S, Margolis B. Mammalian Crumbs3 is a small transmembrane protein linked to protein associated with Lin-7 (Pals1). Gene. 2003;302(1–2):21–9. Epub 2003/01/16. doi: S0378111902010843 [pii]. . 12527193 10.1016/s0378111902010843

[10] Ory DS, Neugeboren BA, Mulligan RC. A stable human-derived packaging cell line for production of high titer retrovirus/vesicular stomatitis virus G pseudotypes. Proc Natl Acad Sci U S A. 1996;93(21):11400–6. Epub 1996/10/15. ; PubMed Central PMCID: PMC38069. 8876147 10.1073/pnas.93.21.11400 PMC38069

[11] Miller-Jensen K, Janes KA, Brugge JS, Lauffenburger DA. Common effector processing mediates cell-specific responses to stimuli. Nature. 2007;448(7153):604–8. Epub 2007/07/20. doi: nature06001 [pii] 10.1038/nature06001 . 17637676 10.1038/nature06001

[12] Vandesompele J, De Preter K, Pattyn F, Poppe B, Van Roy N, De Paepe A, et al Accurate normalization of real-time quantitative RT-PCR data by geometric averaging of multiple internal control genes. Genome Biol. 2002;3(7):RESEARCH0034. Epub 2002/08/20. ; PubMed Central PMCID: PMC126239. 12184808 10.1186/gb-2002-3-7-research0034 PMC126239

[13] Willmarth NE, Ethier SP. Amphiregulin as a novel target for breast cancer therapy. J Mammary Gland Biol Neoplasia. 2008;13(2):171–9. Epub 2008/04/26. 10.1007/s10911-008-9081-9 . 18437539 10.1007/s10911-008-9081-9

[14] Laprise P, Beronja S, Silva-Gagliardi NF, Pellikka M, Jensen AM, McGlade CJ, et al The FERM protein Yurt is a negative regulatory component of the Crumbs complex that controls epithelial polarity and apical membrane size. Dev Cell. 2006;11(3):363–74. Epub 2006/09/05. doi: S1534-5807(06)00257-7 [pii] 10.1016/j.devcel.2006.06.001 ; PubMed Central PMCID: PMC2834949. 16950127 10.1016/j.devcel.2006.06.001 PMC2834949

[15] Wagner KW, Punnoose EA, Januario T, Lawrence DA, Pitti RM, Lancaster K, et al Death-receptor O-glycosylation controls tumor-cell sensitivity to the proapoptotic ligand Apo2L/TRAIL. Nat Med. 2007;13(9):1070–7. Epub 2007/09/04. doi: nm1627 [pii] 10.1038/nm1627 . 17767167 10.1038/nm1627

[16] Stinson S, Lackner MR, Adai AT, Yu N, Kim HJ, O'Brien C, et al TRPS1 targeting by miR-221/222 promotes the epithelial-to-mesenchymal transition in breast cancer. Science signaling. 2011;4(177):ra41 Epub 2011/06/16. 10.1126/scisignal.2001538 . 21673316 10.1126/scisignal.2001538

[17] Hoeflich KP, O'Brien C, Boyd Z, Cavet G, Guerrero S, Jung K, et al In vivo antitumor activity of MEK and phosphatidylinositol 3-kinase inhibitors in basal-like breast cancer models. Clin Cancer Res. 2009;15(14):4649–64. Epub 2009/07/02. doi: 1078-0432.CCR-09-0317 [pii] 10.1158/1078-0432.CCR-09-0317 . 19567590 10.1158/1078-0432.CCR-09-0317

[18] Barretina J, Caponigro G, Stransky N, Venkatesan K, Margolin AA, Kim S, et al The Cancer Cell Line Encyclopedia enables predictive modelling of anticancer drug sensitivity. Nature. 2012;483:603 10.1038/nature11003 https://www.nature.com/articles/nature11003#supplementary-information. 22460905 10.1038/nature11003 PMC3320027

[19] Ince TA, Richardson AL, Bell GW, Saitoh M, Godar S, Karnoub AE, et al Transformation of different human breast epithelial cell types leads to distinct tumor phenotypes. Cancer Cell. 2007;12(2):160–70. Epub 2007/08/19. doi: S1535-6108(07)00179-1 [pii] 10.1016/j.ccr.2007.06.013 . 17692807 10.1016/j.ccr.2007.06.013

[20] Humbert PO, Grzeschik NA, Brumby AM, Galea R, Elsum I, Richardson HE. Control of tumourigenesis by the Scribble/Dlg/Lgl polarity module. Oncogene. 2008;27(55):6888–907. Epub 2008/11/26. doi: onc2008341 [pii] 10.1038/onc.2008.341 . 19029932 10.1038/onc.2008.341

[21] Hariharan IK, Bilder D. Regulation of imaginal disc growth by tumor-suppressor genes in Drosophila. Annu Rev Genet. 2006;40:335–61. Epub 2006/07/29. 10.1146/annurev.genet.39.073003.100738 . 16872256 10.1146/annurev.genet.39.073003.100738

[22] Bilder D, Li M, Perrimon N. Cooperative regulation of cell polarity and growth by Drosophila tumor suppressors. Science. 2000;289(5476):113–6. Epub 2000/07/07. doi: 8658 [pii]. . 10884224 10.1126/science.289.5476.113

[23] Lu H, Bilder D. Endocytic control of epithelial polarity and proliferation in Drosophila. Nat Cell Biol. 2005;7(12):1232–9. Epub 2005/11/01. doi: ncb1324 [pii] 10.1038/ncb1324 . 16258546 10.1038/ncb1324

[24] Moberg KH, Schelble S, Burdick SK, Hariharan IK. Mutations in erupted, the Drosophila ortholog of mammalian tumor susceptibility gene 101, elicit non-cell-autonomous overgrowth. Dev Cell. 2005;9(5):699–710. Epub 2005/11/01. doi: S1534-5807(05)00380-1 [pii] 10.1016/j.devcel.2005.09.018 . 16256744 10.1016/j.devcel.2005.09.018

[25] Hsu YC, Willoughby JJ, Christensen AK, Jensen AM. Mosaic Eyes is a novel component of the Crumbs complex and negatively regulates photoreceptor apical size. Development. 2006;133(24):4849–59. Epub 2006/11/10. doi: dev.02685 [pii] 10.1242/dev.02685 ; PubMed Central PMCID: PMC2836018. 17092952 10.1242/dev.02685 PMC2836018

[26] Nakajima H, Tanoue T. Epithelial cell shape is regulated by Lulu proteins via myosin-II. J Cell Sci. 2010;123(Pt 4):555–66. Epub 2010/01/28. doi: jcs.057752 [pii] 10.1242/jcs.057752 . 20103536 10.1242/jcs.057752

[27] Loie E, Charrier LE, Sollier K, Masson JY, Laprise P. CRB3A Controls the Morphology and Cohesion of Cancer Cells through Ehm2/p114RhoGEF-Dependent Signaling. Molecular and cellular biology. 2015;35(19):3423–35. Epub 2015/07/29. 10.1128/MCB.00673-15 ; PubMed Central PMCID: PMCPmc4561736. 26217016 10.1128/MCB.00673-15 PMC4561736

[28] Nakajima H, Tanoue T. Lulu2 regulates the circumferential actomyosin tensile system in epithelial cells through p114RhoGEF. The Journal of cell biology. 2011;195(2):245–61. Epub 2011/10/19. 10.1083/jcb.201104118 ; PubMed Central PMCID: PMCPmc3198159. 22006950 10.1083/jcb.201104118 PMC3198159

[29] Hashimoto Y, Shindo-Okada N, Tani M, Takeuchi K, Toma H, Yokota J. Identification of genes differentially expressed in association with metastatic potential of K-1735 murine melanoma by messenger RNA differential display. Cancer research. 1996;56(22):5266–71. Epub 1996/11/15. . 8912867

[30] Wang J, Cai Y, Penland R, Chauhan S, Miesfeld RL, Ittmann M. Increased expression of the metastasis-associated gene Ehm2 in prostate cancer. The Prostate. 2006;66(15):1641–52. Epub 2006/08/24. 10.1002/pros.20474 . 16927306 10.1002/pros.20474

[31] Yu H, Ye L, Mansel RE, Zhang Y, Jiang WG. Clinical implications of the influence of Ehm2 on the aggressiveness of breast cancer cells through regulation of matrix metalloproteinase-9 expression. Mol Cancer Res. 2010;8(11):1501–12. Epub 2010/11/05. doi: 1541-7786.MCR-10-0186 [pii] 10.1158/1541-7786.MCR-10-0186 . 21047774 10.1158/1541-7786.MCR-10-0186

[32] Grzeschik NA, Parsons LM, Allott ML, Harvey KF, Richardson HE. Lgl, aPKC, and Crumbs regulate the Salvador/Warts/Hippo pathway through two distinct mechanisms. Curr Biol. 2010;20(7):573–81. Epub 2010/04/07. doi: S0960-9822(10)00151-X [pii] 10.1016/j.cub.2010.01.055 . 20362447 10.1016/j.cub.2010.01.055

[33] Ling C, Zheng Y, Yin F, Yu J, Huang J, Hong Y, et al The apical transmembrane protein Crumbs functions as a tumor suppressor that regulates Hippo signaling by binding to Expanded. Proc Natl Acad Sci U S A. 2010;107(23):10532–7. Epub 2010/05/26. doi: 1004279107 [pii] 10.1073/pnas.1004279107 ; PubMed Central PMCID: PMC2890787. 20498073 10.1073/pnas.1004279107 PMC2890787

[34] Robinson BS, Huang J, Hong Y, Moberg KH. Crumbs regulates Salvador/Warts/Hippo signaling in Drosophila via the FERM-domain protein Expanded. Curr Biol. 2010;20(7):582–90. Epub 2010/04/07. doi: S0960-9822(10)00338-6 [pii] 10.1016/j.cub.2010.03.019 ; PubMed Central PMCID: PMC2855393. 20362445 10.1016/j.cub.2010.03.019 PMC2855393

[35] Chen CL, Gajewski KM, Hamaratoglu F, Bossuyt W, Sansores-Garcia L, Tao C, et al The apical-basal cell polarity determinant Crumbs regulates Hippo signaling in Drosophila. Proc Natl Acad Sci U S A. 2010;107(36):15810–5. Epub 2010/08/28. 10.1073/pnas.1004060107 ; PubMed Central PMCID: PMCPmc2936591. 20798049 10.1073/pnas.1004060107 PMC2936591

[36] Zhao B, Li L, Lei Q, Guan KL. The Hippo-YAP pathway in organ size control and tumorigenesis: an updated version. Genes Dev. 2010;24(9):862–74. Epub 2010/05/05. doi: 24/9/862 [pii] 10.1101/gad.1909210 ; PubMed Central PMCID: PMC2861185. 20439427 10.1101/gad.1909210 PMC2861185

